# Efficacy and Safety of Tegoprazan in *Helicobacter pylori* Eradication: An Umbrella Review of Meta-Analyses

**DOI:** 10.3390/ph19040637

**Published:** 2026-04-17

**Authors:** Dmitrii N. Andreev, Alsu R. Khurmatullina, Igor V. Maev, Dmitry S. Bordin, Andrey V. Zaborovskiy, Yury A. Kucheryavyy, Filipp S. Sokolov, Petr A. Beliy

**Affiliations:** 1 Department of Internal Disease Propaedeutics and Gastroenterology, Russian University of Medicine, 127473 Moscow, Russia; 2 Institute of Clinical Medicine, I. M. Sechenov First Moscow State Medical University (Sechenov University), 119991 Moscow, Russia; 3 Department of Pancreatic, Biliary and Upper Digestive Tract Disorders, A. S. Loginov Moscow Clinical Scientific Center, 111123 Moscow, Russia; 4 Department of General Medical Practice and Family Medicine, Tver State Medical University, 170100 Tver, Russia; 5 Department of Pharmacology, Russian University of Medicine, 127473 Moscow, Russia; 6 Ilyinskaya Hospital, 143421 Krasnogorsk, Russia

**Keywords:** *Helicobacter pylori*, tegoprazan, potassium-competitive acid blocker, eradication, umbrella review, meta-analysis

## Abstract

**Objective:** This umbrella review synthesizes and critically appraises the evidence on the efficacy and safety of tegoprazan-based versus proton pump inhibitor (PPI)-based regimens for *Helicobacter pylori* (*H. pylori*) eradication. **Methods:** This umbrella review was pre-registered in PROSPERO (CRD420251271120). Systematic reviews and meta-analyses published between 1 January 2018 and 10 December 2025 were identified through MEDLINE/PubMed, EMBASE, and the Cochrane Library. Reviews comparing tegoprazan-based and PPI-based eradication regimens in adult patients were included. Methodological quality was assessed using AMSTAR-2, risk of bias with ROBIS, and certainty of evidence with GRADE. Pooled relative risks (RRs) were calculated, with subgroup analyses by study design, treatment duration, and therapeutic regimen. **Results:** Eight systematic reviews and meta-analyses encompassing 17 primary studies and 12,714 participants were included. Tegoprazan-based regimens were associated with a statistically significant improvement in eradication efficacy compared with PPI-based therapies (RR = 1.019; 95% CI: 1.003–1.035; *p* = 0.021). In randomized controlled trials, the benefit was more pronounced (RR = 1.037; 95% CI: 1.015–1.061; *p* = 0.001), whereas no statistically significant benefit was observed in non-randomized studies (RR = 1.014; 95% CI: 0.991–1.037; *p* = 0.235). The efficacy advantage was mainly confined to quadruple therapy regimens (RR = 1.044; 95% CI: 1.002–1.088; *p* = 0.038). Tegoprazan-based regimens were associated with a lower incidence of overall adverse events compared with the PPI group (RR = 0.930; 95% CI: 0.885–0.976; *p* = 0.003). **Conclusions:** Tegoprazan-containing regimens were associated with a modest but statistically significant improvement in *H. pylori* eradication compared with PPI-containing regimens, particularly in randomized controlled trials and quadruple therapy regimens.

## 1. Introduction

*Helicobacter pylori* (*H. pylori*) is a Gram-negative, microaerophilic, spiral-shaped bacterium that colonizes the human gastric mucosa and is associated with the risk of developing chronic gastritis, peptic ulcer disease, and gastric cancer [1,2]. Recent large-scale global and regional data indicate that approximately 43% of the population is infected with *H. pylori* [3,4,5,6]. Eradication therapy, which consists of an antisecretory agent combined with various antibacterial drugs and/or bismuth preparations, is the gold standard of treatment [1,2,7,8]. Recent umbrella reviews (2024–2026) have also shown that several adjunctive or optimization strategies may further improve *H. pylori* eradication outcomes, including probiotic supplementation, rebamipide add-on therapy, and tailored therapy in selected clinical settings [9,10,11]. The successful implementation of these strategies, however, is predicated on the achievement of adequate intragastric pH levels, given that the inhibition of acid production and consequent increase in gastric pH are essential for optimizing the efficacy of ET, as they enhance the concentration and stability of antibiotics [8,12,13].

Potassium-competitive acid blockers (P-CABs) represent a novel class of antisecretory drugs with a mechanism of action based on a reversible ionic interaction with the H^+^, K^+^-ATPase of gastric parietal cells [14,15]. Unlike proton pump inhibitors (PPIs), P-CABs are characterized by a rapid onset of action, stable acid suppression, and independence from CYP2C19 polymorphisms [15,16]. Tegoprazan (C_20_H_19_F_2_N_3_O_3_) is a member of the P-CAB class and is currently used in clinical practice in South Korea, China, India, and Russia [17,18]. Compared to its predecessor, vonoprazan, tegoprazan provides faster and more sustained acid suppression [19,20]. Pharmacokinetic studies indicate a potential synergism between tegoprazan and clarithromycin due to increased systemic exposure of the former; furthermore, tegoprazan may enhance the bioavailability of bismuth preparations [21]. These properties suggest tegoprazan as a potentially preferred antisecretory agent for use in ET regimens.

However, the current evidence on the efficacy and safety of tegoprazan-containing ET regimens compared to PPI-based protocols is considerably heterogeneous. Recent meta-analyses present conflicting findings, ranging from no efficacy advantage of tegoprazan-containing regimens [22] to a marginal increase in efficacy [23]. Moreover, specific comparative data on the efficacy of tegoprazan versus PPIs in eradication regimens of varying duration and composition (e.g., triple, quadruple therapy) are lacking.

The present umbrella review aims to synthesize the existing meta-analyses evaluating the efficacy and safety of tegoprazan-containing versus PPI–based eradication therapies for *H. pylori*, with stratification by therapy regimen (triple and quadruple), treatment duration, and study design.

## 2. Materials and Methods

### 2.1. Study Design and Search Strategy

This study was conducted as an umbrella review in line with the Joanna Briggs Institute guidance for overviews of systematic reviews [24]. This design is particularly suitable when multiple systematic reviews address related clinical questions, because it enables a higher-level synthesis of the existing evidence, evaluation of consistency across reviews, and identification of remaining uncertainties relevant to clinical decision-making and future research [25].

The review protocol was registered prospectively in PROSPERO (CRD420251271120). No deviations from the registered protocol were identified during the conduct of this study. No changes affecting the predefined objectives, eligibility criteria, outcomes, or analytical approach were made after registration. The review was conducted and reported in accordance with the PRISMA 2020 statement [26], and the PRISMA-P checklist is provided in the Supplementary Materials (File S1).

A systematic search was undertaken in MEDLINE/PubMed, EMBASE, and the Cochrane Library. Google Scholar was additionally consulted as a supplementary source to capture potentially missed publications; however, all eligible reviews were verified against the primary databases before inclusion. The search was limited to articles published between 1 January 2018 and 10 December 2025 in order to focus on contemporary evidence.

For PubMed, the search strategy combined Medical Subject Headings and free-text terms related to *H. pylori*, tegoprazan, and evidence synthesis. The search string was as follows: (“Helicobacter pylori”[MeSH] OR “*H. pylori*” OR Helicobacter) AND (“tegoprazan” OR “potassium-competitive acid blocker” OR “P-CAB”) AND (“eradication therapy” OR “combination therapy”) AND (“systematic review” OR “meta-analysis” OR “review”). Equivalent strategies were adapted for the other databases.

### 2.2. Eligibility Criteria and Methodological Quality Assessment

Eligibility was determined according to the PICO framework. The population of interest consisted of adults with confirmed *H. pylori* infection. Reviews focused on animal experiments or in vitro data were not eligible.

The intervention was tegoprazan-containing eradication therapy, whereas comparator regimens consisted of conventional PPI-based eradication strategies or other standard regimens without tegoprazan. The primary efficacy outcome was eradication success, reported as relative risk (RR) or odds ratios (ORs), together with measures of heterogeneity.

We included systematic reviews, with or without meta-analysis, that synthesized evidence from RCTs and/or non-RCTs. Narrative reviews, case reports, retrospective descriptive studies, and reviews based solely on observational non-comparative evidence were excluded. No language restrictions were applied.

Reviews were also excluded if they did not report a predefined protocol, lacked a sufficiently comprehensive search strategy, omitted formal risk-of-bias assessment, failed to provide extractable quantitative outcome data, did not clearly define the target population or intervention, lacked a direct comparison between tegoprazan-based and standard regimens, or reported outcomes inadequately.

Methodological quality was appraised independently by two reviewers (F.S.S. and P.A.B.) using a modified AMSTAR-2 tool [27]. The 16 domains of the instrument were rated as “yes”, “partial yes”, or “no”. Agreement between reviewers was assessed using Cohen’s kappa and percentage agreement before consensus, with kappa values interpreted as strong (>0.7), moderate (0.5–0.7), or low (<0.5).

### 2.3. Risk of Bias and Certainty of Evidence Assessment

Risk of bias in the included systematic reviews was assessed using the ROBIS tool [28], which evaluates concerns across four domains: study eligibility criteria, identification and selection of studies, data collection and appraisal, and synthesis and findings.

Each domain was judged as low risk, some concerns, or high risk of bias. The certainty of evidence for each outcome was then evaluated using the GRADE approach [29].

These assessments were performed independently by two reviewers (F.S.S. and A.Z.V.), and disagreements were resolved through discussion with a third reviewer (P.A.B.). ROBIS traffic-light plots were generated to provide a visual summary of risk-of-bias judgments.

### 2.4. Assessment of Overlap Among Primary Studies

To determine the degree of overlap between the included systematic reviews, we applied the Graphical Representation of Overlap for OVErviews (GROOVE) approach [30]. An evidence matrix was constructed to identify shared and unique primary studies, and the corrected covered area (CCA) was calculated to quantify overlap.

### 2.5. Study Selection and Data Extraction

Two reviewers (A.R.K. and D.N.A.) independently performed study selection and data extraction, with Y.A.K. acting as the third reviewer when needed. During the first stage, titles, abstracts, and keywords were screened for potential relevance. Full-text eligibility assessment was then carried out by D.S.B. and A.V.Z. Any disagreements were resolved through discussion, with F.S.S. involved when consensus could not be reached initially.

Data were extracted using a predefined standardized form. For each eligible review, we recorded the year of publication, number of included primary studies, clinical context, characteristics of tegoprazan-based and comparator regimens, total sample size, effect measures, number of successful eradication events, and statistical methods used. Additional variables included treatment line, AEs, and overall eradication outcomes.

### 2.6. Statistical Analysis

Where necessary, reported effect measures were recalculated as RRs. For efficacy outcomes, RR values greater than 1 were interpreted as favoring tegoprazan. For AEs, RR values greater than 1 indicated a higher event risk in the tegoprazan group.

Because clinical and methodological differences between studies were expected, the choice of pooling model was based on statistical heterogeneity. A fixed-effects model was used when heterogeneity was low (I^2^ ≤ 50%), whereas a random-effects model was applied when heterogeneity exceeded 50%. Heterogeneity was quantified using the I^2^ statistic.

Prespecified subgroup analyses were undertaken according to study design, duration of eradication therapy, and regimen type. AE analyses were performed on pooled data from the meta-analyses. These analyses were intended to explore potential sources of heterogeneity and evaluate the stability of the observed treatment effects across clinically relevant settings.

All effect estimates are presented with 95% confidence intervals, and a two-sided *p* value < 0.05 was considered statistically significant. Statistical analyses were performed using RevMan version software (version 5.4.1; London, UK).

## 3. Results

### 3.1. Study Selection

A total of 312 records were identified through electronic database searches. After removal of 74 duplicate records, 238 unique articles were screened based on titles and abstracts. During this stage, 186 records were excluded because they did not evaluate tegoprazan-based regimens, or were not relevant to *H. pylori* eradication therapy.

The full texts of the remaining 52 articles were retrieved and assessed for eligibility. Of these, 44 studies were excluded for the following reasons: lack of a direct comparison between tegoprazan-containing and standard eradication regimens (n = 27), insufficient quantitative data to extract effect estimates (n = 11), or insufficient methodological rigor (n = 6).

**Figure 1:**
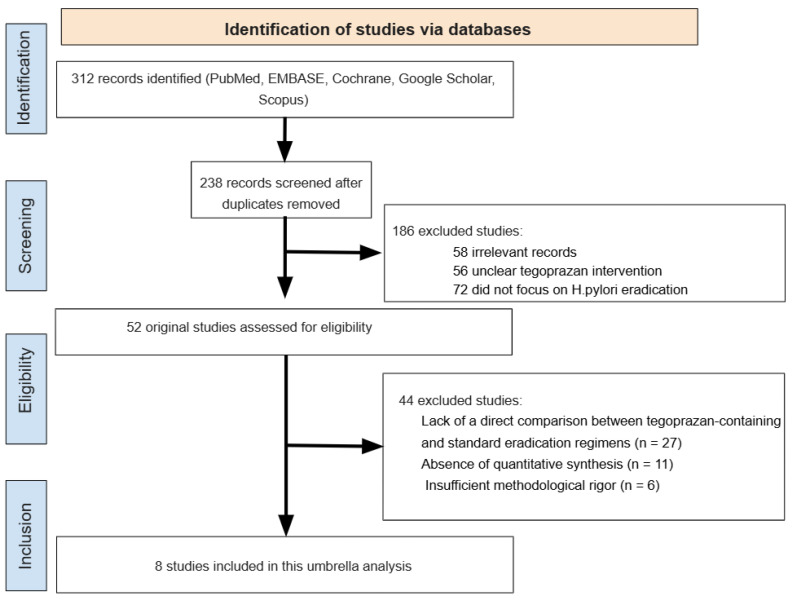
PRISMA (Preferred Reporting Items for Systematic Reviews and Meta-Analyses) flow diagram.

Ultimately, 8 systematic reviews and meta-analyses met all the inclusion criteria and were included in the umbrella review. These reviews collectively evaluated the efficacy and safety of tegoprazan-based regimens for *H. pylori* eradication across different treatment lines (Figure 1).

Table 1 presents an overview of the principal characteristics of the included systematic reviews and meta-analyses, including their primary outcomes, total sample sizes, tegoprazan-based interventions, comparator regimens, as well as the corresponding risk-of-bias judgments and overall quality assessments. The certainty of evidence varied across outcomes. Analyses restricted to randomized controlled trials (RCTs) were generally supported by moderate- to high-certainty evidence, whereas mixed-design meta-analyses were more frequently rated as low to moderate certainty. These differences should be considered when interpreting the pooled estimates.

### 3.2. ROBIS Assessment

An overview of the risk-of-bias evaluation conducted using the ROBIS tool is provided in Supplementary File S1. Among the systematic reviews examining tegoprazan-based regimens for *H. pylori* eradication, the greatest concerns regarding bias were consistently observed within the study eligibility criteria and identification and selection of studies domain. In contrast, the data collection and study appraisal and synthesis and findings domains demonstrated the lowest levels of bias across the included reviews.

Overall, the ROBIS assessment indicated that most reviews applied clearly defined inclusion criteria and comprehensive search strategies, while limitations were primarily related to inconsistencies in outcome assessment, appraisal of primary studies, and transparency of data extraction procedures.

### 3.3. GROOVE Analysis

A total of 17 primary studies, encompassing 12,714 participants, were identified across the included systematic reviews and meta-analyses evaluating tegoprazan-containing eradication regimens. The degree of overlap among primary studies was assessed using the GROOVE methodology.

For tegoprazan use in *H. pylori* eradication therapy, the corrected covered area (CCA) was calculated at 26.53%, indicating a very high level of overlap among the included reviews. Adjustment for chronological structural missingness yielded comparable values, suggesting stable and consistent evidence coverage.

Graphical representations of the GROOVE analyses are presented in Supplementary File S2, illustrating the extent of overlap across reviews and supporting the robustness of the summarized evidence base.

### 3.4. Effectiveness of Tegoprazan

**Figure 2:**
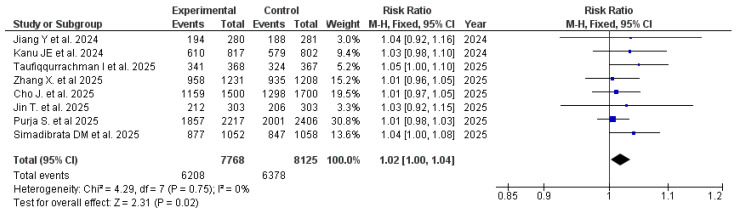
RR for *H. pylori* eradication rates in regimens including tegoprazan versus standard therapy including PPIs [22,23,31,32,33,34,35,36].

The pooled analysis demonstrated a statistically significant advantage of tegoprazan-based *H. pylori* eradication regimens compared with standard PPI-based regimens, with a pooled RR of 1.019 (95% CI: 1.003–1.035; *p* = 0.021). No heterogeneity was observed among the included studies (I^2^ = 0%), indicating a high level of consistency; therefore, a fixed-effects model was applied. Figure 2 demonstrates the consistency of effect estimates across the included meta-analyses (RRs across outcomes), with no observed heterogeneity (I^2^ = 0%), supporting the robustness of the pooled estimate.

To further evaluate treatment effectiveness, we compared the pooled prevalence of successful eradication achieved with tegoprazan-based regimens versus comparator regimens (Table 2). Across all included studies (n = 8 meta-analyses), the pooled eradication rate in the tegoprazan group was 79.22% (95% CI: 74.88–83.24), compared with 77.05% (95% CI: 72.95–80.91) in the comparator group.

### 3.5. Subgroup Analysis

#### 3.5.1. Study Design

Separate subgroup analyses were conducted for RCTs and non-randomized studies (non-RCTs). In non-RCTs, the pooled effect was not statistically significant (RR = 1.014; 95% CI: 0.991–1.037; *p* = 0.235). In contrast, RCTs demonstrated a statistically significant improvement in eradication rates with the addition of tegoprazan (RR = 1.037; 95% CI: 1.015–1.061; *p* = 0.001), indicating a more robust treatment effect in high-quality randomized evidence.

#### 3.5.2. Treatment Duration

Eradication outcomes were also analyzed according to treatment duration. For 7-day regimens, the pooled RR was 1.047 (95% CI: 0.963–1.138; *p* = 0.284), which did not reach statistical significance. Similarly, 14-day regimens showed no significant difference (RR = 1.004; 95% CI: 0.977–1.031; *p* = 0.800).

#### 3.5.3. Therapeutic Regimen

Further analyses were performed based on the type of eradication regimen. In triple therapy regimens, the pooled RR was 1.009 (95% CI: 0.979–1.039; *p* = 0.573), indicating no significant improvement. However, in quadruple therapy regimens, the addition of tegoprazan resulted in a statistically significant benefit (RR = 1.044; 95% CI: 1.002–1.088; *p* = 0.038).

**Figure 3:**
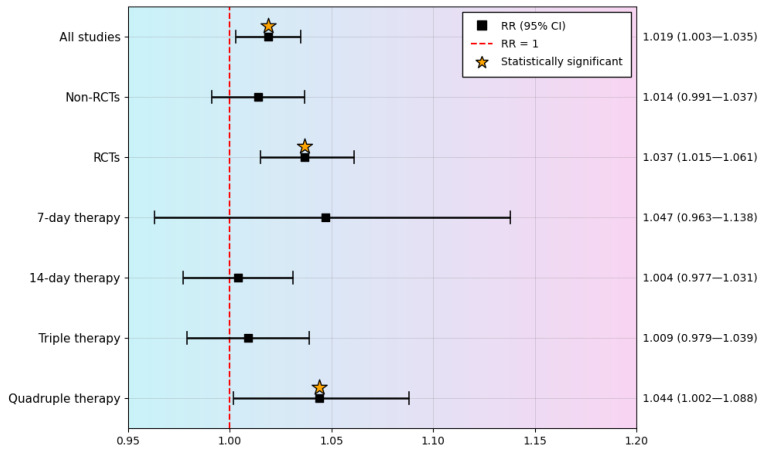
Forest plot summarizing pooled RRs.

A comprehensive forest plot was constructed to visually summarize the pooled RRs across all included studies, displaying the comparative efficacy of tegoprazan-based therapies. Figure 3 stratifies the results by therapy type, treatment duration, and study design, providing a clear overview of both effectiveness and safety across the different regimens.

#### 3.5.4. Adverse Events

Safety outcomes were assessed by comparing the incidence of overall adverse events (AEs) between tegoprazan-based regimens and comparative regimens. In the pooled analysis of AEs, the incidence was 34.4% in the tegoprazan-based group and 37.1% in the PPI group, corresponding to a RR of 0.930 (95% CI: 0.885–0.976; *p* = 0.003), indicating a favorable safety profile without an increased risk of treatment-related AEs.

## 4. Discussion

### 4.1. Main Findings

This study was designed as an umbrella review of systematic reviews and meta-analyses addressing tegoprazan-based versus PPI-based eradication regimens in adult patients with *H. pylori* infection. Only reviews with explicit comparative quantitative data and adequate methodological transparency were included. This design allowed us to synthesize the highest currently available level of evidence while also assessing methodological quality, overlap, and certainty of evidence.

In this umbrella review, we synthesized evidence from eight systematic reviews and meta-analyses evaluating tegoprazan-containing regimens for *H. pylori* eradication. The pooled analysis showed a modest but statistically significant improvement in eradication efficacy with tegoprazan compared with PPI-based regimens (RR = 1.019; 95% CI: 1.003–1.035; *p* = 0.021). The pooled eradication rate was 79.2% in the tegoprazan group and 77.1% in the PPI group. In analyses restricted to RCTs, the benefit was more pronounced (RR = 1.037; 95% CI: 1.015–1.061; *p* = 0.001), whereas no statistically significant benefit was observed in non-RCTs (RR = 1.014; 95% CI: 0.991–1.037; *p* = 0.235). Subgroup analyses suggested that the apparent advantage of tegoprazan was mainly confined to quadruple therapy regimens (RR = 1.044; 95% CI: 1.002–1.088; *p* = 0.038), while no significant superiority was demonstrated in triple therapy. Notably, a high-quality meta-analysis with low risk of bias, including six studies, likewise reported a statistically significant improvement in eradication efficacy with tegoprazan compared with PPIs [23], supporting the consistency of the observed effect. From a safety perspective, tegoprazan-based regimens were associated with a lower overall AE rate than PPI-based regimens. In the pooled analysis, overall adverse events were reported in 34.4% of patients in the tegoprazan group and 37.1% in the PPI group, corresponding to an RR of 0.930 (95% CI: 0.885–0.976; *p* = 0.003). Importantly, improved acid suppression was not associated with an increased burden of treatment-related AEs, which is a key consideration in real-world eradication strategies.

These findings are best interpreted in light of the well-established concept that the degree and stability of gastric acid suppression critically determine the success of *H. pylori* eradication therapy. Suppression of gastric acid increases intragastric pH, which in turn enhances both the chemical stability and antibacterial activity of commonly used antibiotics [37]. Experimental studies have shown that amoxicillin and clarithromycin are significantly more stable in gastric juice at neutral pH, with a marked prolongation of their effective half-life as pH approaches 7.0 [38]. This pharmacokinetic effect directly translates into higher intragastric antibiotic exposure during eradication therapy.

The clinical relevance of this principle has been repeatedly confirmed in observational studies, RCTs, and meta-analyses long before the introduction of P-CABs. Data from the European Registry on *H. pylori* management (Hp-EuReg) clearly demonstrate that higher doses and longer effective exposure to PPIs significantly improve eradication rates across a wide range of regimens [39]. Earlier meta-analyses have consistently shown that high-dose PPIs outperform standard-dose regimens in triple therapy [40,41].

Tegoprazan represents an extension of established pharmacological logic. As a P-CAB, tegoprazan provides faster onset and more stable acid suppression compared with conventional PPIs, with minimal influence from CYP2C19 polymorphisms [42,43]. These characteristics are particularly relevant during the early phase of eradication therapy, when rapid elevation of intragastric pH may maximize antibiotic efficacy from the first day of treatment [15]. The effect size observed in this umbrella review is therefore consistent with the known pharmacological effects of stronger acid suppression.

Antibiotic resistance in *H. pylori* has become a major global challenge, with multiple recent meta-analyses demonstrating steadily increasing resistance rates to key antibiotics across diverse regions, undermining the efficacy of standard eradication regimens [44,45,46,47]. The MIC90 values of key antibiotics against *H. pylori*, including penicillin’s, clarithromycin, and tetracycline, decrease substantially at higher pH levels, particularly around pH 7.0–7.5 [48]. Therefore, more profound and sustained acid inhibition effectively lowers the pharmacodynamic threshold required for bacterial eradication. This advantage translates into clinically meaningful benefits in the setting of antibiotic resistance: meta-analytic evidence suggests that P-CAB-based regimens achieve significantly higher eradication rates than PPI-based therapies in patients infected with clarithromycin-resistant *H. pylori* (73.7% vs. 41.5%; RR = 1.53; 95% CI: 1.07–2.20; *p* = 0.02), implying a potential clinical benefit in the setting of antibiotic resistance [33].

The finding that the benefit of tegoprazan was more evident in quadruple therapy regimens is of particular interest. Quadruple therapies, especially those including bismuth, rely on multiple synergistic mechanisms, and optimized acid suppression may enhance both antibiotic activity and bismuth bioavailability [2,21]. In contrast, the absence of a clear advantage in triple therapy or shorter regimens suggests that acid suppression alone may be insufficient to overcome other limiting factors, most notably antibiotic resistance [49].

From a pharmacological perspective, the favorable safety profile of P-CABs may be related to their rapid, reversible, and more predictable inhibition of gastric acid secretion, which avoids excessive acid suppression beyond the treatment window [50]. In contrast to concerns derived from long-term PPI use in other indications, short-course eradication therapy does not appear to amplify safety risks with stronger acid inhibition [51]. Collectively, the available evidence suggests that tegoprazan-based regimens offer an improved or at least comparable tolerability profile relative to PPIs, reinforcing their suitability for short-term *H. pylori* eradication without introducing additional safety concerns [9].

### 4.2. Strengths and Limitations

This umbrella review has notable strengths. It integrates the highest level of synthesized evidence, applies rigorous methodological quality assessment using AMSTAR-2, ROBIS, and GRADE frameworks, and systematically evaluates overlap among primary studies using the GROOVE methodology. Stratified analyses by study design, treatment duration, and therapeutic regimen provide an understanding of where tegoprazan may offer the greatest benefit.

However, several limitations should be acknowledged. The corrected covered area indicated a very high degree of overlap among primary studies, reflecting a relatively limited pool of original RCTs repeatedly included across meta-analyses. Additionally, heterogeneity in regimen composition, geographic regions, and antibiotic resistance patterns may limit generalizability. Another limitation relates to the methodological quality of the included reviews. ROBIS assessment identified recurrent concerns in study eligibility criteria and identification/selection of studies, which may have introduced bias into the underlying evidence base and should be considered when interpreting the pooled estimates. An additional explanation for the modest effect size observed in this umbrella review is that the currently available comparative evidence for tegoprazan is derived almost exclusively from East Asian populations. This may be important because the efficacy of PPI-based eradication therapy is influenced by CYP2C19 genotype: extensive metabolizers have lower cure rates than carriers of loss-of-function alleles, and reduced-function CYP2C19 phenotypes are markedly more common in East Asian individuals than in European individuals [52]. Therefore, the incremental advantage of tegoprazan over PPIs may be attenuated in East Asian studies, whereas in European populations it could theoretically be greater. Although direct tegoprazan data in Europe are currently lacking, the phase III PHALCON-HP trial conducted in the United States and Europe demonstrated superior overall eradication rates for vonoprazan-based regimens versus lansoprazole-based triple therapy, and the 2024 ACG guideline now includes P-CAB dual therapy as a suitable empiric alternative [2,53].

Despite these limitations, this umbrella review provides the first structured and comprehensive synthesis of the available evidence on tegoprazan-based therapies.

## 5. Conclusions

The findings of this umbrella review suggest that tegoprazan-based regimens provide a modest but statistically significant improvement in *H. pylori* eradication compared with PPI-based therapy, particularly in RCTs and quadruple therapy regimens. Tegoprazan-based regimens were also associated with a lower overall rate of AEs.

## Figures and Tables

**Table 1:** Included Studies.

Author, Year	Number of Included Studies, Types of Included Studies	Type of Tegoprazan Regimen	Type of Comparison Regimen	Number of Included Patients	Number of Effectively Treated Patients	Number of Included Controls	Number of Effectively Treated Controls	Effect Measure	Quality Evaluation, AMSTAR-2	Quality Evaluation, GRADE
Jiang Y et al. 2024 [31]	2 RCTs	Tegoprazan-based triple regimen	PPI-based triple regimen	175	110	175	106	RR 1.67 (95% CI: 1.38–2.01)	Low	Low
		Tegoprazan-based quadruple regimen	PPI-based quadruple regimen	105	84	106	82	RR 1.03 (95% CI: 0.90–1.19)		
Kanu JE et al. 2024 [32]	2 RCTs, 2 non-RCTs	Tegoprazan-based triple regimen	PPI-based triple regimen	817	610	802	802	RR 1.03 (95% CI: 0.98–1.10)	Moderate	Moderate
Jin T. et al. 2025 [33]	4 RCTs	Tegoprazan-based triple regimen	PPI-based quadruple regimen	303	212	303	206	RR 1.03 (95% CI: 0.92–1.15)	High	Moderate
Cho J. et al. 2025 [34]	3 RCTs and 4 non-RCTs	Tegoprazan-based triple and quadruple regimen	PPI-based triple and quadruple regimen	1500	1159	1700	1298	RR 1.01 (95% CI: 0.97–1.05)	Moderate	Low
Purja S. et al. 2025 [22]	3 RCTs and 6 non-RCTs	Tegoprazan-based triple regimen	PPI-based triple regimen	1018	765	1222	921	RR 1.00 (95% CI: 0.95–1.05)	Moderate	Moderate
		Tegoprazan-based quadruple regimen	PPI-based quadruple regimen	193	152	186	140	RR 1.05 (95% CI: 0.94–1.17)		
		Tegoprazan-based triple and quadruple regimen in Clarithromycin-Resistant Strains, including tegoprazan	PPI-based triple and quadruple regimen in Clarithromycin-Resistant Strains	43	15	32	5	RR 2.23 (95% CI: 0.91–5.51)		
Simadibrata DM et al. 2025 [23]	6 RCTs	Tegoprazan-based triple and quadruple regimen	PPI-based triple and quadruple regimen	1052	877	1058	847	RR 1.05 (95% CI: 1.01–1.08)	High	High
Taufiqqurrachman I. et al. 2025 [35]	2 RCTs	Tegoprazan-based quadruple regimen	PPI-based quadruple regimen	368	341	367	324	RR 1.05 (95% CI: 1.00–1.10)	Moderate	Low
Zhang X. et al. 2025 [36]	8 RCTs	Tegoprazan-based dual regimen	PPI-based dual regimen	184	158	184	155	RR 1.02 (95% CI: 0.94–1.11)	High	High
		Tegoprazan-based triple regimen	PPI-based triple regimen	954	716	927	698	RR 1.00 (95% CI: 0.95–1.05)		
		Tegoprazan-based bismuth quadruple regimen	PPI-based quadruple regimen	93	84	97	82	RR 1.07 (95% CI: 0.96–1.19)		

**Table 2:** Comparison of Included Studies.

Type of Comparison, n of Included Meta-Analysis	RR (95% CI)	Pooled Tegoprazan Efficiency (%) and 95% CI	Pooled PPI Efficiency (%) and 95% CI	*p*-Value for RR
All studies, 8	1.019 (95% CI: 1.003–1.035)	79.22 (95% CI: 74.88–83.24)	77.05 (95% CI: 72.95–80.91)	0.021
Non-RCTs, 4	1.014 (95% CI: 0.991–1.037)	76.41 (95% CI: 75.13–78.18)	74.73 (95% CI: 72.08–77.29)	0.235
RCTs, 8	1.037 (95% CI: 1.015–1.061)	80.05 (95% CI: 78.83–81.22)	75.98 (95% CI: 71.59–78.26)	0.001
7-day therapy, 2	1.047 (95% CI: 0.963–1.138)	67.42 (95% CI: 59.06–75.26)	64.62 (95% CI: 57.50–71.43)	0.284
14-day therapy, 4	1.004 (95% CI: 0.977–1.031)	77.83 (95% CI: 73.43–81.93)	76.91 (95% CI: 71.93–81.55)	0.800
Triple therapy, 4	1.009 (95% CI: 0.979–1.039)	73.19 (95% CI: 69.79–76.46)	72.02 (95% CI: 67.82–76.04)	0.573
Quadruple therapy, 5	1.044 (95% CI: 1.002–1.088)	82.90 (95% CI: 72.14–91.46)	78.91 (95% CI: 69.68–86.85)	0.038

## Data Availability

No new data were created or analyzed in this study. Data sharing is not applicable to this article.

## Supplementary Materials

The following supporting information can be downloaded at https://www.mdpi.com/article/10.3390/ph19040637/s1. File S1. Completed PRISMA-P checklist. File S2: ROBIS assessment; Graphical visualizations of the GROOVE assessment.
