# Supplementary material for: Efficacy and Safety of Tegoprazan in Helicobacter pylori Eradication: An Umbrella Review of Meta-Analyses
Source: Pharmaceuticals (Basel). 2026 Apr 17;19(4):637. doi: 10.3390/ph19040637 (PMC13118615; doi:10.3390/ph19040637)
Supplement: Supplementary file 1 [file pharmaceuticals-19-00637-s001.zip › File S2.pdf]

Supplementary 1. ROBIS assessment

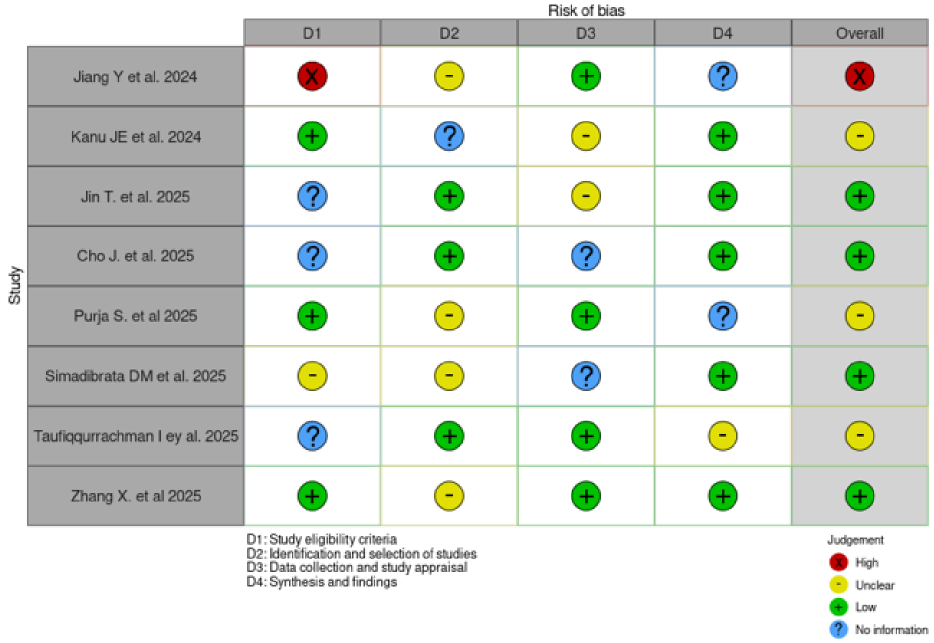

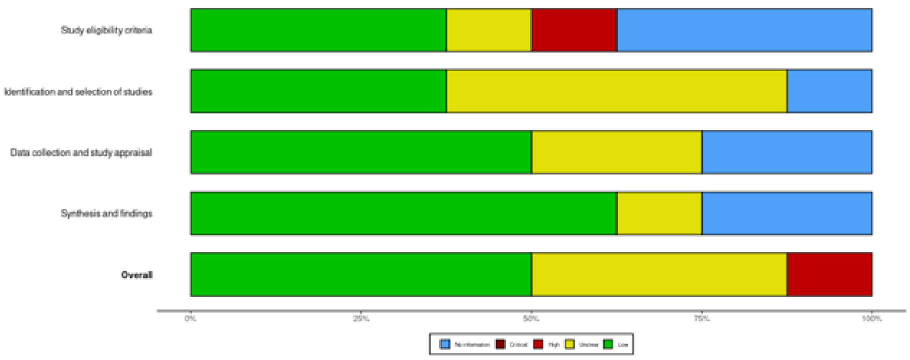

Supplementary 1. GROOVE assessment

	Jin 2025	Kanu JE et al. 202	Jiang Y et al. 2024	Cho J. et al. 2025	Purja S. et al 2025	Simadibrata DM et al. 2025	Taufiqqurrachman I ey al. 2025
Kanu JE et al. 2024	33.3%						
Jiang Y et al. 2024	50.0%	50.0%					
Cho J. et al. 2025	22.2%	37.5%	28.6%				
Purja S. et al 2025	22.2%	57.1%	28.6%	75.0%			
Simadibrata DM et al. 2025	25.0%	25.0%	33.3%	30.0%	30.0%		
Taufiqqurrachman I ey al. 2025	20.0%	20.0%	33.3%	12.5%	12.5%	33.3%	
Zhang X. et al 2025	9.1%	33.3%	11.1%	50.0%	66.7%	40.0%	0.0%

**28**	=	**Total nodes (pairs of reviews)**
1	=	Slight overlap (<5%)
1	=	Moderate overlap (5% to <10%)
3	=	High overlap (10% to <15%)
23	=	Very High overlap (≥15%)
